# Targeting senescent cells to boost bone fracture healing

**DOI:** 10.1172/JCI181974

**Published:** 2024-06-17

**Authors:** Lorenz C. Hofbauer, Ulrike Baschant, Christine Hofbauer

**Affiliations:** 1 Division of Endocrinology, Diabetes and Bone Diseases, Department of Medicine III & University Center for Healthy Aging, Technische Universität Dresden Medical Center, Dresden, Germany.; 2 Center for Regenerative Therapies Dresden, Technische Universität Dresden, Dresden, Germany.; 3 Division of Endocrinology, Orthopedic, Trauma and Plastic Surgery Center, Technische Universität Dresden Medical Center, Dresden, Germany,; 4 Nationales Zentrum für Tumorerkrankungen (NCT/UCC), Technische Universität Dresden Medical Center, Dresden, Germany.

## Abstract

Bone fracture healing is a complex process with distinct phases: the inflammatory phase, the soft and hard callus formation, and the remodeling phase. In older individuals, bone healing can be delayed or disturbed, leading to non-union fractures at worst. The initial healing phases require communication between immune cells and osteoprogenitor cells. However, senescence in these cell types impedes fracture healing by unknown mechanisms. In this issue of the *JCI*, Saul et al. showed that two distinct senescent p21-expressing cell populations, an osteochondroprogenitor cell and a neutrophil subpopulation, intrinsically impair fracture healing in mice irrespective of age. Genetic ablation of p21-positive cells accelerated fracture healing, while removal of a different senescent cell population, p16-positive cells, made no difference. Conceptually, this view of senescence in fracture healing with a spotlight on osteoimmune cross-talk provides a promising rationale for therapies to boost bone repair at all ages.

## Fracture healing under pressure

Bone fracture healing is a complex regenerative process and essential for resuming and maintaining mobility after trauma. There are two distinct types of bone fracture healing: primary bone healing, an intramembranous process without the formation of a periosteal callus, and secondary bone healing, an endochondral regenerative process that takes place after internal or external fixation of the fracture (1). Provided that there is adequate stability and sufficient vascular supply, four distinct, yet partially overlapping phases can be defined, named after the predominant biological characteristics: (a) the acute inflammatory phase, (b) soft callus formation and (c) hard callus formation in the repair period, and (d) the remodeling phase (1). The inflammatory phase starts immediately after the tissue trauma with hematoma formation and is triggered by hematoma-associated growth factors and cytokines that recruit and attract proinflammatory immune cells and osteochondroprogenitor cells. The soft callus phase is characterized by the formation of a granulation tissue, including new vessels and cartilage resulting from the activities of mesenchymal cells, fibroblasts, and endothelial cells after proper resolution of the inflammation. Angiogenesis is key in this phase, and the soft callus includes chondrogenesis producing a collagen-rich fibrocartilaginous network at the fracture ends. This process creates a scaffold of limited stability. In the hard callus phase, the soft callus is being resorbed and replaced by a bone matrix produced by osteoblasts that gradually becomes calcified and provides greater stability (1). The transition from cartilage to immature bone is termed endochondral ossification and similarly takes place during longitudinal growth of long bones, for instance at the femur and the tibia. In the remodeling phase, the immature bone produced by endochondral ossification is subsequently reshaped to its original contour and biomechanical functionality by slow and coupled cycles of osteoclastic bone resorption followed by osteoblastic bone formation that may take years (1).

With an aging population, declining muscle force and poor bone strength coincide with falls and fractures that cluster at a time in life when comorbidities, e.g., vascular diseases, diabetes mellitus, and rheumatoid arthritis, jeopardize normal fracture healing and restoration of the health status prior to the fracture (2). Common features of these entities include chronic and unchecked inflammation, impaired vascular supply with tissue hypoxia, a higher vulnerability for infections, and reduced recruitment and differentiation of osteoprogenitor cells toward osteoblasts, as are present in patients with diabetes mellitus (3). Clinical complications of delayed or incomplete fracture healing may be epitomized in non-union fracture, a severe and debilitating condition. Delayed fracture healing occurs more often in the elderly for the reasons discussed above; however, the role of aging, i.e., senescent cells, in delayed fracture has remained largely unclear.

## Senescent cells in bone are the enemy within

Aging is accompanied by increased appearance of senescent cells, but senescent cells are also present following tissue injury, even at a young age. Senescent cells have only recently been implicated in the pathogenesis of bone diseases, mainly at the level of the osteocyte. Osteocytes are mechanosensing cells embedded in the bone mineral that are derived from the osteoblast lineage and display a neuron-like shape, forming a syncytium that communicates within the bone tissue via gap junctions. The senescence phenotype in osteocytes is established by activation of cyclin-dependent kinase inhibitors p16^Ink4a^ (p16) and p21^Cip1^ (p21), resulting in DNA double-strand breaks as well as a senescence-associated secretory phenotype (SASP), creating a proinflammatory microenvironment that promotes bone loss. Investigators from the Mayo Clinic implicated senescent osteocytes in bone loss associated with aging (4), periodontal infection (5), radiation (6), and diabetes mellitus (7). These studies focused mainly on p16-positive cells and indicated that genetic or pharmacological senolytic strategies could mitigate age-related bone loss (4).

**Figure 1:**
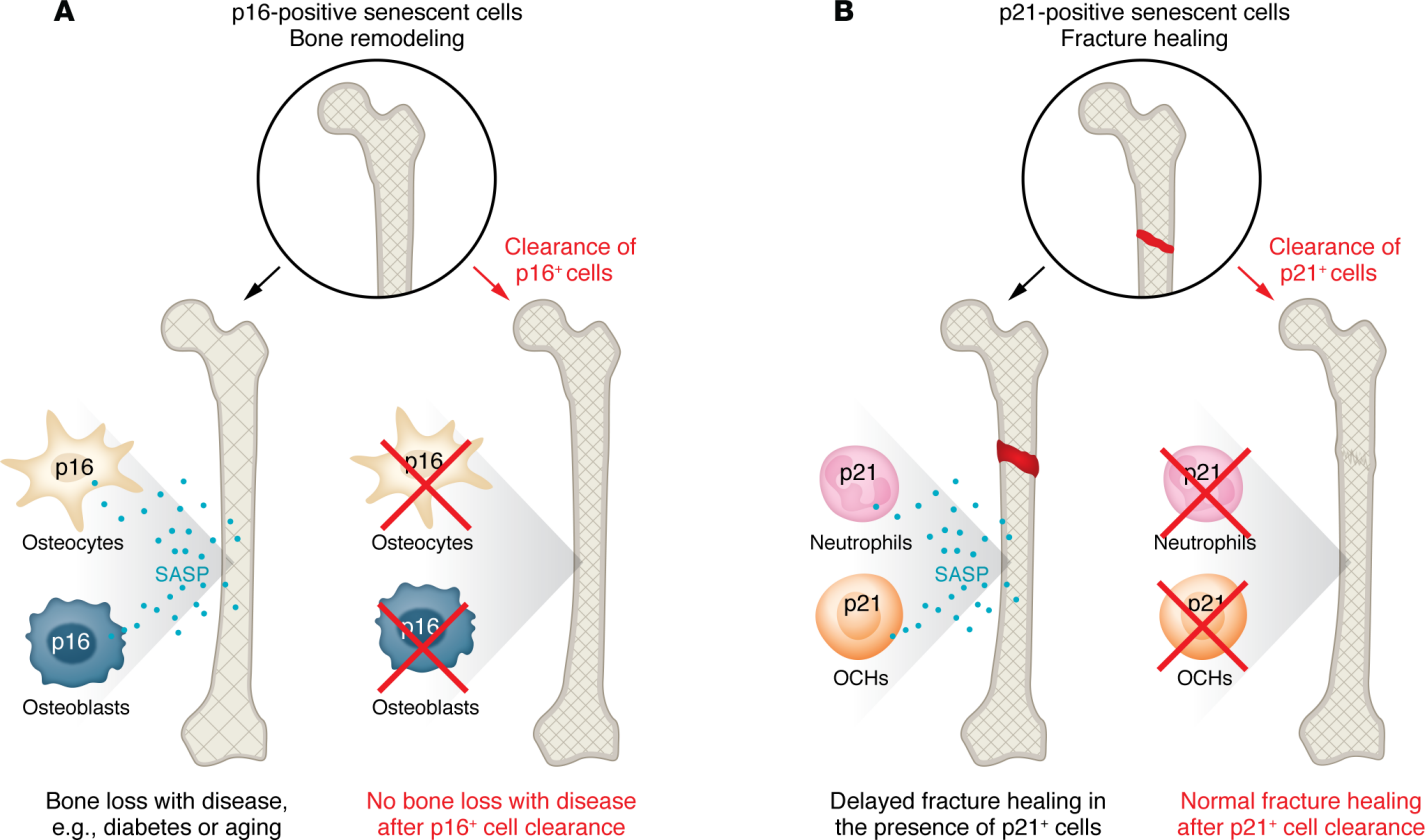
p16- and p21-positive skeletal cells have diverse roles in models of bone loss and fracture healing. (**A**) Senescent bone cells, osteocytes and osteoblasts, expressing p16 contribute to bone loss associated with aging, diabetes mellitus, and other diseases. Clearance of p16-positive cells mitigates bone loss. (**B**) After fracture, p21-expressing osteochondroprogenitor (OCH) cells and neutrophils delay fracture healing by promoting a proinflammatory microenvironment and by inhibiting bone formation. Clearance of p21-expressing cells accelerates fracture healing. SASP, senescence-associated secretory phenotype.

In this issue of the *JCI*, Saul et al. (8) discovered a mechanism by which cellular senescence may also impede fracture repair. Employing young and old mice after skeletal injury that created a tibial fracture, the authors characterized cellular senescence in the fracture area based on the expression of p16 or p21. Cells expressing p16 or p21 were shown to increase during age-related bone loss (4). However, their abundance pattern in the callus after fracture was different. While p16-positive cells increased only at a later stage of fracture healing, there was a rapid, but transient, peak of p21-positive cells immediately after the skeletal injury (8). Intriguingly, the p21-positive cells consisted of osteochondroprogenitor cells and neutrophils. When p21-positive cells were cleared by a targeted genetic model that the authors had previously developed, the *p21-ATTAC* model (6), senescence expression profiles within the fracture callus microenvironment were suppressed and the fracture healed faster irrespective of age. However, this intervention did not affect age-related bone loss. In the *p21-ATTAC* mouse, the *p21^Cip1^* promoter drives a “suicide” transgene that encodes an inducible caspase-8, which selectively kills p21^Cip1^-expressing senescent cells when induced. By contrast, clearance of p16-positive cells with a similar approach, the *p16^Ink4a^-ATTAC* model, did not affect fracture healing, while it mitigated age-related bone loss. Functional proteomic characterization of the p21-positive neutrophils revealed their potential to induce stromal senescence in a paracrine manner, whereas p21-positive osteochondroprogenitor cells displayed signaling pathways known to inhibit bone formation. Intriguingly, p21-positive osteochondroprogenitor cells showed a stem cell–like proinflammatory profile, with shared features of a fibroadipogenic progenitor population that appeared after muscle injury, indicating a broader role for these cells to control tissue repair. This finding is important, since bone and muscle injury often occur together after trauma or surgery in humans, for instance after proximal femoral fracture following a fall. Saul et al. clearly dissect the inhibitory role of two distinct senescent cell types, the p16-positive cells for age-related bone loss and the p21-positive cells for fracture repair, thus allowing a more specific therapeutic interference (Figure 1) (8).

## Toward therapies for fracture healing

Saul et al. (8) elegantly provide a refined view of the two p21-positive cell populations, neutrophils and osteochondroprogenitors, and their cross-talk in the early phase of bone fracture healing that is a key regulatory mechanism of tissue repair. The findings provide a robust mechanistic basis for further research and future therapeutic development. If the intention is to enhance fracture healing, then p21-positive senescent cells are now a clearly marked target for senolytic therapies. Interfering with p21-positive cells may in fact boost fracture healing across all ages. It remains to be seen whether this strategy is sufficient to accelerate bone fracture healing also in disorders that typically come with delayed fracture healing in humans, including diabetes mellitus of glucocorticoid excess. Another open question relates to the evolutionary function of these injury-specific p21-positive proinflammatory cells that inhibit bone and muscle tissue repair. One possibility is that this inhibitory relay prevents excessive tissue formation in an attempt to restore the form-follows-function principle of design.

Currently, no osteoporosis drug has been clinically approved to enhance fracture healing, although they are commonly used to treat patients with osteoporotic fractures and markedly reduce future fracture risk. Conceptually, antiresorptives inhibit osteoclasts in the remodeling phase, thus interfering only late in the last phase of the fracture healing process. Even bone-anabolic therapies, such as parathyroid receptor agonists or sclerostin-neutralizing antibodies, have limited efficacy in bone defect regeneration — which shares some mechanisms with fracture healing — in normal and diabetic rodents (9, 10), although they stimulate bone formation and yield the greatest gain of bone mass and strength in rodents and humans (9–11). However, their effects on the phases of fracture healing may be limited, since they mainly promote osteogenesis in the callus formation and remodeling phases, but have only a minimal effect on the initial inflammatory phase. This first phase may be only targeted by therapies that interfere with senescent cells and their osteoimmune dialogue. Such therapies have the prospect of benefitting many patients with bone fractures and muscle injuries, young and old.

## Version 1 06/17/2024

Electronic publication

## References

[1] Einhorn TA, Gerstenfeld LC (2015). Fracture healing: mechanisms and interventions. Nat Rev Rheumatol.

[2] Wildemann B (2021). Non-union bone fractures. Nat Rev Dis Primers.

[3] Hofbauer LC (2022). Bone fragility in diabetes: novel concepts and clinical implications. Lancet Diabetes Endocrinol.

[4] Farr JN (2017). Targeting cellular senescence prevents age-related bone loss in mice. Nat Med.

[5] Aquino-Martinez R (2021). Senescent cells exacerbate chronic inflammation and contribute to periodontal disease progression in old mice. J Periodontol.

[6] Chandra A (2020). Targeted reduction of senescent cell burden alleviates focal radiotherapy-related bone loss. J Bone Miner Res.

[7] Eckhardt BA (2020). Accelerated osteocyte senescence and skeletal fragility in mice with type 2 diabetes. JCI Insight.

[8] Saul D (2024). Osteochondroprogenitor cells and neutrophils expressing p21 and senescence markers modulate fracture repair. J Clin Invest.

[9] Hamann C (2013). Sclerostin antibody treatment improves bone mass, bone strength, and bone defect regeneration in rats with type 2 diabetes mellitus. J Bone Miner Res.

[10] Hamann C (2014). Effects of parathyroid hormone on bone mass, bone strength, and bone regeneration in male rats with type 2 diabetes mellitus. Endocrinology.

[11] Cosman F (2016). Romosozumab treatment in postmenopausal women with osteoporosis. N Engl J Med.

